# Chloro-1,4-dimethyl-9*H*-carbazole Derivatives Displaying Anti-HIV Activity

**DOI:** 10.3390/molecules23020286

**Published:** 2018-01-30

**Authors:** Carmela Saturnino, Fedora Grande, Stefano Aquaro, Anna Caruso, Domenico Iacopetta, Maria Grazia Bonomo, Pasquale Longo, Dominique Schols, Maria Stefania Sinicropi

**Affiliations:** 1 Department of Science, University of Basilicata, 85100 Potenza, Italy; carmela.saturnino@unibas.it (C.S.); mariagrazia.bonomo@unibas.it (M.G.B.); 2 Department of Pharmacy, Health & Nutritional Sciences, University of Calabria, 87036 Arcavacata di Rende, Italy; anna.caruso@unical.it (A.C.); domenico.iacopetta@unical.it (D.I.); s.sinicropi@unical.it (M.S.S.); 3 Department of Chemistry and Biology, University of Salerno, 84084 Fisciano, Italy; plongo@unisa.it; 4 KU Leuven, Rega Institute for Medical Research, Herestraat 49, B-3000 Leuven, Belgium; dominique.schols@kuleuven.be

**Keywords:** carbazole derivatives, HIV, antiviral agents

## Abstract

Background: Despite the progress achieved by anti-retroviral drug research in the last decades, the discovery of novel compounds endowed with selective antiviral activity and reduced side effects is still a necessity. At present, the most urgent requirement includes the improvement of HIV (Human Immunodeficiency Virus) prevention and sexual transmission and the development of new drugs to treat the chronic lifelong infection. Methods: Six chloro-1,4-dimethyl-9*H*-carbazoles (**2a**,**b**–**4a**,**b**) have been prepared following opportunely modified known chemical procedures and tested in luciferase and *Escherichia coli* β-galactosidase expressing CD4^+^, CXCR4^+^, CCR5^+^ TZM-bl cells. Results and Conclusion: a preliminary biological investigation on the synthesized small series of chloro-1,4-dimethyl-9*H*-carbazoles has been carried out. Among all tested compounds, a nitro-derivative (**3b**) showed the most interesting profile representing a suitable lead for the development of novel anti-HIV drugs.

## 1. Introduction

Nowadays, according to the National Institute on Drug Abuse (NIDA), part of the National Institutes of Health, approximately 37 million people are infected with HIV worldwide. HIV, the etiologic agent of AIDS (Acquired Immune Deficiency Syndrome), is a retrovirus belonging to the subclass of lentiviruses that thus shows an RNA genome, which guarantees a higher genetic variability and rapid adaptability [1].

At present, 25 anti-HIV drugs targeting reverse transcriptase, protease, integrase and viral entry and a pharmacokinetic enhancer are available. The most clinically adopted treatment consists in a combination of three anti-HIV drugs from at least two different classes in order to inhibit viral replication and diminish the onset of drug resistance [2]. This approach has resulted in a significant decrease of viral replication in HIV-infected individuals as well as a reduction of the risk of viral transmission [3,4]. Despite the encouraging results of the most recent treatments, in a considerable number of cases a therapeutic failure occurred mostly due to the virus capability to remain in a quiescent form in infected cells without being completely eradicated [5]. Considering the unremitting spread of HIV along with unpredictable outbreak of old or new virus strains, it is desirable to possess an arsenal of suitable countermeasures in order to prevent global health crises and/or to strive to control the further spreading. 

At present, at least two sub-types of HIV are known: HIV-1 and HIV-2. Type 1 is able to integrate into the host genome (mostly in memory CD4^+^ T cells) that can harbor latent HIV-1 DNA for many years, and is characterized by dynamic genetic diversity which generates variants or recombinant forms [6,7]. HIV-2, at first isolated in Africa, is less infectious and has a tendency to develop slowly giving rise to a milder infection [8]. Several studies over the last years have demonstrated that HIV-entry is mediated, not only by CD4 receptor, but also by members of the chemokine receptor family, in particular, CC-chemokine receptor 5 (CCR5) and CXC-chemokine receptor 4 (CXCR4), play a key role [9].

The HIV capability of utilizing these chemokine-receptors is essential in the viral tropisms: CXCR4 (expressed on T cells) is usually involved in cellular entry of T-tropic (X4) HIV-1 strains, while M-tropic (R5) HIV-1 entry is generally mediated by CCR5 (expressed on monocytes-macrophages), while further viral strains are able to use both receptors (dual-tropic) [10].

Thus, the research focusing on the discovery of novel compounds gifted with antiviral activity is still very attractive and challenging [11,12,13,14,15,16,17]. It is essential to develop effective treatment strategies for people affected by this devastating infection. At present, the most promising approaches include an improvement of HIV prevention through innovative gene therapies as well as the development of new small-molecule drugs to treat the infection [18,19,20,21,22].

Although many drugs from synthetic sources are commercially available, some natural compounds have shown interesting antiviral properties that could be optimized, for instance, by chemical modifications aimed to increase the efficacy and/or selectivity, diminishing side effects.

In this scenario, some alkaloids or analogues bearing a carbazole scaffold have been shown to be very effective, both in in vitro and in vivo assays, as tools for the treatment of several acute or chronic diseases [9,23,24,25,26,27,28,29,30] and, as well, exhibited antiviral activity [31,32,33].

Considering that in many cases, a number of limitations including drug resistance, severe side effects, and long-term treatment may be adducted as explanations for the clinical failure of the commercial anti-HIV drugs, we have been attracted from the idea to explore the anti-HIV activity of carbazole derivatives. Herein, we report a preliminary biological investigation of a small series of chloro-1,4-dimethyl-9*H*-carbazoles recently prepared by our research group [34,35,36,37] as potential anti-HIV drugs candidates. One of these compounds showed an interesting activity and thus could be amenable to additional structural modifications for an enhanced potency. Further structural modifications will be reported in due course.

## 2. Results and Discussion

A small series of six chloro-1,4-dimethyl-9*H*-carbazoles (**2a**,**b**–**4a**,**b**) has been prepared following opportunely modified chemical procedures previously reported [37]. 

**Scheme 1:**
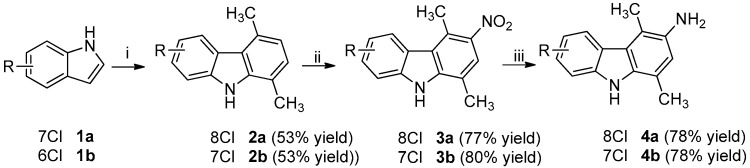
Synthesis of chloro-1,4-dimethyl-9*H*-carbazoles. Reagents and conditions: (i) acetonylacetone, *p*-TSA, ethanol, 6 h, reflux; (ii) HNO_3_, acetic anhydride, CH_2_Cl_2_, 5 min, −15 °C; (iii) CH_3_CO_2_H/HCl, SnCl_2_, DMF, 3 h, 100 °C.

Briefly, starting from the commercially available indoles **1a**–**b**, the corresponding carbazole derivatives **2a**–**b** were synthesized according to the method of Cranwell and Saxton [38]. These intermediates were transformed into the nitro derivatives **3a**–**b** that were in turn reduced by stannous chloride to furnish the 3-amino-1,4-dimethyl-9*H*-carbazoles **4a**–**b** with good yields (Scheme 1) [39].

In order to understand their mechanism of action, these compounds have been tested in CD4^+^, CXCR4^+^, CCR5^+^ TZM-bl cells [40] and some of them showed a moderate antiviral activity, although no significant differences have been observed when compounds were tested against CXCR4- or CCR5-using viruses (Table 1). These results suggest that the antiviral activity is probably also due to the inhibition of a different stage of HIV replication cycle. 

The obtained outcomes led us to the following remarks. The compounds bearing as R substituent a chlorine at position 7 (**2**–**4b**) showed the greater activity against HIV. On the contrary, the compounds functionalized with the chlorine at position 8 (**2**–**4a**) showed only a moderate antiviral activity, indicating that the position of the halogen plays a crucial role in the preservation of the activity. In particular, the nitro compound **3b** showed the higher activity against HIV demonstrating that not only the chlorine position on the carbazole moiety is crucial for activity but it can be improved by the concomitant presence of an electro-attractor group. In conclusion, among all tested compounds, the 7-chloro-3-nitro derivative **3b** resulted in the most promising compound and warrants further investigation.

## 3. Materials and Methods 

Commercial reagents were purchased from Aldrich (Saint-Quentin Fallavier, France), Acros Organics (Geel, Belgium), and Alfa Aesar (Schiltigheim, France) and used without additional purification. Flash chromatography was performed on Merck (Merck Frankfurt, Germany) silica gel (0.040–0.063 mm). Melting points were determined in open capillary tubes on a Büchi 535 electrothermal apparatus (BÜCHI, Switzerland) and are uncorrected. ^1^H and ^13^C spectra were registered on a Bruker AC 300 or Bruker AC 600 (Bruker Biospin, Rheinstetten, Germany) using CDCl_3_ as solvent, unless otherwise stated. Chemical shifts are reported in ppm. The abbreviations used are as follows: s, singlet; d, doublet; dd, double doublet; bs, broad signal. MS spectrometry analysis was carried out on a Finnigan LCQ Deca-Ion Trap Instrument (San Jose, CA, USA). The elemental analyses for C, H, N were recorded on a Thermo Finnigan Flash EA 1112 series (Waltham, MA, USA) and performed according to standard microanalytical procedures.

### 3.1. General Procedure for the Preparation of 1,4-Dimethyl-9H-carbazole Derivatives *(**2a**–**b**)*

Acetonylacetone (0.70 mL, 6.00 mmol) and *p*-toluenesulphonic acid were added dropwise to a stirred solution of indole derivative **1a**–**b** (6.00 mmol) in ethanol (10 mL). This reaction mixture was maintained under reflux for 6 h and then concentrated in vacuo. The crude product was purified by flash chromatography.

#### 3.1.1. 8-Chloro-1,4-dimethyl-9*H*-carbazole (**2a**)

Column chromatography of the residue on silica gel (ethyl acetate/*n*-hexane 1:9→3:7) gave **2a** (53% yield) as a white solid. ^1^H-NMR (300 MHz, CDCl_3_): δ 10.50 (s, 1H, NH); 7.50 (d, 1H, Ar); 7.39 (d, 1H, Ar); 7.27 (d, 2H, Ar); 7.16-7.08 (t, 1H, Ar); 2.82 (s, 6H, CH_3_). ^13^C-NMR (75 MHz, CDCl_3_): δ 140.13, 138.98, 129.98, 125.54, 124.02, 123.97, 121.21, 120.45, 119.76, 119.54, 119.23, 117.65, 18.87, 16.56. FAB-MS *m*/*z*: 230 [M + H]^+^. Anal. Calcd. for C_14_H_12_ClN: C, 73.20; H, 5.27; N, 6.10. Found: C, 73.18; H, 5.29; N, 6.11.

#### 3.1.2. 7-Chloro-1,4-dimethyl-9*H*-carbazole (**2b**)

Column chromatography of the residue on silica gel (ethyl acetate/*n*-hexane 1:9→3:7) gave 2b (50% yield) as a white solid. ^1^H-NMR (300 MHz, CDCl_3_): δ 10.50 (s, 1H, NH); 7.50 (d, 1H, Ar); 7.42 (s, 1H, Ar); 7.27 (d, 2H, Ar); 7.16-7.05 (t, 1H, Ar); 2.82 (s, 6H, CH_3_). ^13^C-NMR (75 MHz, CDCl_3_): δ 140.11, 137.98, 129.99, 128.32, 125.98, 123.99, 123.87, 121.45, 121.23, 120.43, 117.23, 111.98, 18.76, 16.64. FAB-MS *m*/*z*: 230 [M + H]^+^. Anal. Calcd. for C_14_H_12_ClN: C, 73.20; H, 5.27; N, 6.10. Found: C, 73.19; H, 5.25; N, 6.09.

### 3.2. General Procedure for the Preparation of 3-Nitro-1,4-dimethyl-9H-carbazole Derivatives *(**3a**–**b**)*

To a cooled (−15 °C) solution of 1,4-dimethyl-9*H*-carbazole derivative **2a**–**b** (4.35 mmol) in 20 mL of dichloromethane was added dropwise a solution of acetic anhydride (7.25 mL) and fuming nitric acid (d = 1.52 g/mL, 0.27 mL). After 5 min, the reaction mixture was poured into crushed ice; then, pH solution was adjusted at 9 with 1 M solution of NaOH. After filtration, the crude product was purified by flash chromatography.

#### 3.2.1. 8-Chloro-1,4-dimethyl-3-nitro-9*H*-carbazole (**3a**)

Column chromatography of the residue on silica gel (chloroform) and crystallization with ethanol/water 1:1 gave 3a (77% yield) as a white solid. m.p. > 250 °C. ^1^H-NMR (300 MHz, CDCl_3_): δ 10.60 (s, 1H, NH); 7.74 (d, 1H, Ar); 7.60 (s, 1H, Ar); 7.44 (d, 2H, Ar); 2.65 (s, 6H, CH_3_). ^13^C-NMR (75 MHz, CDCl_3_): δ 142.32, 140.21, 138.87, 124.56, 121.98, 120.56, 119.98, 119.87, 119.76, 118.43, 115.43, 102.32, 15.54, 14.13. FAB-MS *m*/*z*: 275 [M + H]^+^. Anal. Calcd. for C_14_H_11_ClN_2_O_2_: C, 61.21; H, 4.04; N, 10.20. Found: C, 61.20; H, 4.03; N, 10.18.

#### 3.2.2. 7-Chloro-1,4-dimethyl-3-nitro-9*H*-carbazole (**3b**)

Column chromatography of the residue on silica gel (chloroform) and crystallization with ethanol/water 1:1 gave 3b (80% yield) as a white solid. m.p. > 250 °C. ^1^H-NMR (300 MHz, CDCl_3_): δ 10.60 (s, 1H, NH); 7.74 (d, 1H, Ar); 7.60 (s, 1H, Ar); 7.44 (s, 1H, Ar); 7.01 (d, 1H, Ar); 2.65 (s, 6H, CH_3_). ^13^C-NMR (75MHz, CDCl_3_): δ 142.22, 140.21, 136.98, 128.22, 123.98, 121.87, 121.32, 120.47, 118.46, 115.32, 111.98, 102.54, 15.56, 14.15. FAB-MS *m*/*z*: 275 [M + H]^+^. Anal. Calcd. for C_14_H_11_ClN_2_O_2_: C, 61.21; H, 4.04; N, 10.20. Found: C, 61.19; H, 4.02; N, 10.21.

### 3.3. General Procedure for the Preparation of 3-Amino-1,4-dimethyl-9H-carbazole Derivatives *(**4a**–**b**)*

A stirred solution of 3-nitro-1,4-dimethyl-9*H*-carbazole derivatives **3a**–**b** (3.6 mmol) in *N*,*N*-dimethylformamide (2 mL) was heated at 100 °C. The resulting solution was poured into a mixture containing 37% hydrochloride acid (2.6 mL), acetic acid (0.78 mL) and stannous chloride (18 mmol). The solution was stirred at 100 °C for 3 h and cooled to room temperature. The obtained suspension was poured into 20% stirred solution of sodium hydroxide and filtered. The solid was washed with water, air-dried and purified by flash chromatography.

#### 3.3.1. 8-Chloro-1,4-dimethyl-3-amino-9*H*-carbazole (**4a**)

Column chromatography of the residue on silica gel (ethyl acetate) and crystallization with ethanol gave 4a (78% yield) as a white solid. m.p. 198 °C. ^1^H-NMR (300 MHz, CDCl_3_): δ 10.68 (s, 1H, NH); 7.50 (d, 1H, Ar); 7.35 (d, 1H, Ar); 7.10 (d, 1H, Ar); 6.88-6.83 (t, 1H, Ar); 4.04 (d, 2H, NH_2_); 2.65 (s, 6H, CH_3_). ^13^C-NMR (75 MHz, CDCl_3_): δ 140.65, 140.54, 138.98, 124.23, 120.54, 119.87, 119.54, 119.23, 118.89, 113.34, 106.98, 102.43, 16.76, 12.34. FAB-MS *m*/*z*: 245 [M + H]^+^. Anal. Calcd. for C_14_H_13_ClN_2_: C, 68.71; H, 5.35; N, 11.45. Found: C, 68.70; H, 5.33; N, 11.46.

#### 3.3.2. 7-Chloro-1,4-dimethyl-3-amino-9*H*-carbazole (**4b**)

Column chromatography of the residue on silica gel (ethyl acetate) and crystallization with ethanol gave 4b (78% yield) as a white solid. m.p. 200 °C. ^1^H-NMR (300 MHz, CDCl_3_): δ 10.68 (s, 1H, NH); 7.50 (d, 1H, Ar); 7.10 (d, 1H, Ar); 6.88 (t, 1H, Ar); 5.82 (d, 1H, Ar); 4.04 (d, 2H, NH_2_); 2.65 (s, 6H, CH_3_). ^13^C-NMR (75 MHz, CDCl_3_): δ 140.55, 140.14, 136.98, 128.23, 123.57, 121.32, 120.54, 118.21, 113.32, 111.98, 106.98, 102.21, 16.56, 12.23. FAB-MS *m*/*z*: 245 [M + H]^+^. Anal. Calcd. for C_14_H_13_ClN_2_: C, 68.71; H, 5.35; N, 11.45. Found: C, 68.72; H, 5.33; N, 11.47.

### 3.4. Anti-HIV-1 Replication Assay

The firefly luciferase and *Escherichia coli* β-galactosidase expressing CD4^+^, CXCR4^+^, CCR5^+^ TZM-bl cells (50 μL; 2 × 10^5^ cells/mL) were resuspended in cell culture medium supplemented with 15 μg/ml diethylaminoethyl-dextran (DEAE-Dextran; Sigma-Aldrich, Diegem, Belgium) and pre-incubated for 30 min at 37 °C in 96-well plates with in cell culture medium diluted test compounds (100 μL). Next, the T-tropic (X4) HIV-1 strain NL4.3 or the M-tropic (R5) HIV-1 BaL was added (in 50 μL) according to the TCID50 of the viral stock. As control compounds AMD3100 (CXCR4 inhibitor) and maraviroc (CCR5 inhibitor) were included. Two days post-infection, viral replication is measured by luminescence. Steadylite plus reagent (Perkin Elmer, Zaventem, Belgium) was mixed with lyophilized substrate according to manufacturer’s guidelines. Supernatant (120 μL) was removed and 75 μL Steadylite plus substrate solution was added to the 96-well plates. Next, the plates were incubated in dark for 10 min in a closed plate shaker (PHMP, Grant, Shepreth, Cambridgeshire, UK). Finally, cell lysis was scored microscopically and 100 μL supernatant was transferred to white 96-well plates (Greiner Bio-One, Frickenhausen, Germany) to measure the relative luminescence units (RLUs) using the SpectraMax L microplate reader and Softmax Pro software (Molecular Devices, Sunnyvale, CA, USA) with an integration time of 0.6 s and dark adapt of 5 min.

## 4. Conclusions

The preliminary biological study of a small series of chloro-1,4-dimethyl-9*H*-carbazoles for their anti-HIV-1 activity is here reported. Among all tested compounds, the nitro-derivative **3b** showed the most interesting profile indicating that the chlorine position on the carbazole scaffold could be important for its antiviral activity. In particular, the R_1_ substituent seems to affect the antiviral activity in the following order: R_1_ = H < NH_2_ < NO_2_. Overall, the obtained data indicate that this class of compounds represents suitable starting points for the development of selective and perhaps alternative tools for the treatment of chronic infections induced by HIV.

## Figures and Tables

**Table 1:** Anti-HIV-1 activity profile of compounds evaluated in TZM-bl cells.

Compound		IC_50_ [μM]		CC_50_ [μM]	S.I.
		NL4.3 X4	Bal R5		
**2a**	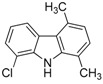	19.2	30.6	13.6	No
**2b**	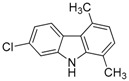	3.5	3.8	7.3	2
**3a**	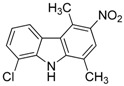	n.d.	n.d.	n.d.	n.d.
**3b**	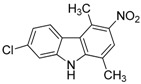	1.4	5.3	22.7	4–16
**4a**	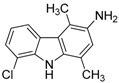	10.2	12.1	14.7	1
**4b**	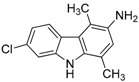	9.3	9.5	37.9	4
**AMD3100**	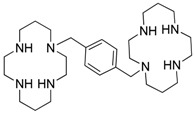	0.95 × 10^−3^	>1000	>1000	
**Maraviroc**	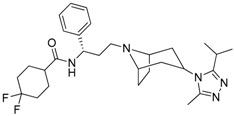	>1000	4.2	>1000	

IC_50_: Inhibitory Concentration 50%; CC_50_: Cytotoxicity Concentration 50%; S.I. Selectivity Index calculated by CC_50_/IC_50_; nd: not done. The cytotoxicity was investigated by cell viability tests using the Trypan blue dye exclusion method.
